# A fine-tuned global distribution dataset of marine forests

**DOI:** 10.1038/s41597-020-0459-x

**Published:** 2020-04-14

**Authors:** Jorge Assis, Eliza Fragkopoulou, Duarte Frade, João Neiva, André Oliveira, David Abecasis, Sylvain Faugeron, Ester A. Serrão

**Affiliations:** 1 CCMAR – Centre of Marine Sciences, University of Algarve, 8005-139 Faro, Portugal; 2 Centro de Conservación Marina and CeBiB, Facultad de Ciencias Biológicas, Pontificia Universidad Católica de Chile, Santiago, Chile; 3 UMI 3614 Evolutionary Biology and Ecology of Algae, CNRS, Sorbonne Université, Pontificia Universidad Católica de Chile, Universidad Austral de Chile, Station Biologique, Roscoff, France

**Keywords:** Biogeography, Ecological modelling, Climate-change ecology, Biodiversity

## Abstract

Species distribution records are a prerequisite to follow climate-induced range shifts across space and time. However, synthesizing information from various sources such as peer-reviewed literature, herbaria, digital repositories and citizen science initiatives is not only costly and time consuming, but also challenging, as data may contain thematic and taxonomic errors and generally lack standardized formats. We address this gap for important marine ecosystem-structuring species of large brown algae and seagrasses. We gathered distribution records from various sources and provide a fine-tuned dataset with ~2.8 million dereplicated records, taxonomically standardized for 682 species, and considering important physiological and biogeographical traits. Specifically, a flagging system was implemented to signal potentially incorrect records reported on land, in regions with limiting light conditions for photosynthesis, and outside the known distribution of species, as inferred from the most recent published literature. We document the procedure and provide a dataset in tabular format based on Darwin Core Standard (DwC), alongside with a set of functions in R language for data management and visualization.

## Abstract (summary)

**Table:** 

Measurement(s)	Species • Distribution
Technology Type(s)	digital curation
Factor Type(s)	geographic location
Sample Characteristic - Organism	Fucales • Laminariales • Tilopteridales • Cymodoceaceae • Hydrocharitaceae • Posidoniaceae • Zosteraceae
Sample Characteristic - Environment	marine biome
Sample Characteristic - Location	global

Machine-accessible metadata file describing the reported data: 10.6084/m9.figshare.12011523

## Background & Summary

Bioclimatic modelling^1,2^, macroecology^3^ and evolution^4^ are fields that have recently seen a boost in broad scale analyses owing to increased accessibility of large scale biodiversity data. Although these can be obtained from digital online databases (e.g., GBIF, the Global Biodiversity Information Facility, www.gbif.org and OBIS, the Ocean Biogeographic Information System, www.obis.org), herbarium (e.g., Macroalgal Herbarium Portal, www.macroalgae.org), museum collections, as well as citizen science initiatives^5–7^, they can be very incomplete and contain geographical and taxonomic errors. In particular, studies focused on the impacts of global climate changes^8,9^, or locating evolutionary biodiversity hotspots^10,11^, require complete and extremely accurate baselines on the distribution of species across space and time^12^.

Collating broad-scale biodiversity data from multiple sources is challenged by two major obstacles. First, the lack of complete database compatibility allowing efficient information exchange between distinct sources, alongside with inconsistent file structures^13,14^, leaves data frequently scattered, even for well‐known taxa^15^. Second, the quality of several sources has been questioned regarding potential geographical data errors^16^. This is a serious limitation since unreliable biased records can deeply influence the outcomes of research analyses. For instance, distribution models can be strongly influenced by particular marginal records. While records of marine species falling on land (and vice-versa) can be easily identified and dealt with^10^, those distributed in climatically unfavorable regions (i.e., outside species’ niche), beyond range margins or dispersal capacities, should be verified and corrected when necessary. Wrong records may be even more likely for rare, elusive, or cryptic species that can be easily confused with others, more common and broadly distributed^17^. An additional problem that is more evident and easier to tackle is related to taxonomic data errors^16^, which can deeply confound the baseline of a species’ distribution^18^. When properly reviewed, databases can integrate quality control flags to identify potential data limitations. While some research communities have developed quality control standards on data (e.g., The Ocean Data Standards and Best Practices Project, www.oceandatastandards.org), no implementation has been done so far for the aforementioned data limitations, even in major online data sources providing large scale biodiversity data.

Here we provide a fine-tuned dataset of marine forests at global scales, with occurrence records gathered from numerous independent sources^19,20^ and flagged with automatic and manual pipelines to increase data reliability in terms of geographical (including depth) and taxonomical traits. “Marine forests” is a common name used here to designate large brown algae (kelp and fucoids) and seagrasses. These blue-green infrastructures rank among the most productive and biodiversity-rich ecosystems^21^, supporting diverse food webs^22,23^, critical habitats and nursery grounds for numerous associated species^24,25^. They increase local biodiversity levels^23,25–27^ and provide key ecological services^21^ such as nutrient cycling, carbon sequestration^28,29^, sediment stabilization, and natural protection against ocean wave energy^23^. Because climate change is shifting their distribution and abundance worldwide^1,8,30,31^, a comprehensive dataset providing essential baselines is needed to better report and understand marine forests’ variability across space and time^14^.

## Methods

### Data compilation

Occurrence records of marine forests of large brown algae (orders Fucales, Laminariales and Tilopteridales), and seagrasses (families Cymodoceaceae, Hydrocharitaceae, Posidoniaceae and Zosteraceae) were gathered from online repositories and herbaria, peer-reviewed scientific literature and citizen science initiatives with independently verifiable data (e.g., supported by photos). Only records with no copyright for any use and without any restriction (e.g., CC0, www.creativecommons.org), or any use with appropriate attribution (e.g., CC BY), were stored in the dataset (please refer to the analytical list of data sources; Suppl. Table 1).

### Data treatment

The dataset structure was based on Darwin Core Standard (DwC)^32^. This framework for biodiversity data offers a stable and flexible framework to store all fields available in original data sources. Moreover, it provides standard identifiers, labels, and definitions, allowing a full link-back to original data sources.

Taxonomic standardization was performed with the World Register of Marine Species (WoRMS; www.marinespecies.org), a universally authoritative open-access reference system for marine organisms. This tool provides a unique identifier (aphiaID) that enabled to link each taxon originally captured, to an internationally accepted standardized name with associated taxonomic information (including hierarchy, rank, acceptance status and synonymy) that will continue to be updated in the future in case of taxonomic or name changes. In the rare cases of no match with WoRMS (including misspelled entries), or uncertain taxonomic status, the records were removed from the dataset.

Geographical locations were available for most records as coordinates in decimal degrees. For those records missing coordinates, but including information on location, an automatic geocoding procedure was performed with OpenStreetMap^33,34^ service (http://planet.openstreetmap.org).

Since unique records may be available across distinct data sources, the final aggregated dataset was subjected to the removal of duplicate records. These were considered when belonging to the same taxon, and recorded in the same exact geographical location (longitude, latitude and depth) and date (year, month and day).

### Quality control

To achieve a fine-tuned dataset, a flagging system was implemented to identify records with doubtful geographical and depth locations. This started by flagging records occurring on land, by using a 1 km threshold from shoreline. This distance represented the lower spatial resolution of the polygon used to define landmass (OpenStreetMap geographic information^33^). Light availability for photosynthesis was further considered, since it is the main environmental driver restricting the vertical distribution of marine forests^35^. Limiting light was favored in detriment of bathymetry, because it varies with depth throughout the global ocean, particularly in oceanic regions, were it reaches deeper waters^1^. Available light at bottom was extracted from Bio-ORACLE^36^, a dataset providing benthic environmental layers (i.e., along the bottom of the ocean). Because Bio-ORACLE layers are available for 3 different depth ranges, the maximum light value per record was chosen as a conservative approach to estimate the potential depth range for a given location. Records were flagged when light values were below the known limiting threshold of 50 E.m^−2^.year^−1^ for marine forests’ photosynthesis^35,37^. This flag was not applied to the brown algae *Sargassum fluitans*, *Sargassum natans*^38^ and *Sargassum pusillum*^39^ as they can complete a full life cycle floating on the sea surface.

Finally, all records were manually verified to identify potential outliers outside the known distribution of species. This information was based on the most recent published literature and by consulting experts when possible. Because distributional ranges are often documented at an administrative level (e.g., country), the flagging procedure integrated the Marine Ecoregions of the World (MEOW)^40^, a scheme that represents the broad-scale distributional patterns of species/communities in the ocean^40^. Records were flagged when distributed in a MEOW region not considered in the information available in the literature or provided by experts. The MEOW has 3 distinct levels dividing the globe into 12 realms, 62 provinces and 232 ecoregions^40^. We adopted the intermediate level “provinces” to reduce commission errors (cases incorrectly identified as potential outliers) and omission errors (outliers left out, or omitted), potentially arising while considering “realms” and “ecoregions”, respectively. Records were removed from the database when no information was available in literature to support the actual distribution of species.

## Data Records

The dataset is publicly accessible for download in a permanent Figshare^41^ repository (10.6084/m9.figshare.7854767). A version containing only pruned records is also accessible at https://www.dataone.org and https://www.marineforests.com.

### Taxonomic coverage

**Fig. 1:**
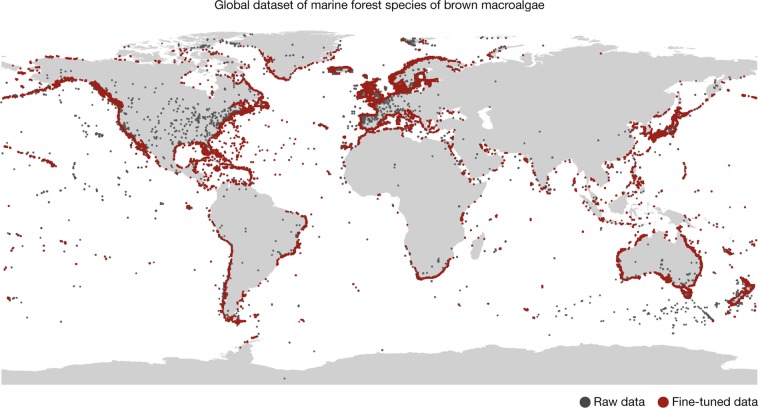
Global dataset of marine forest species of brown macroalgae. Included orders: Fucales, Laminariales and Tilopteridales. Red and gray circles depict raw and corrected data, respectively.

**Fig. 2:**
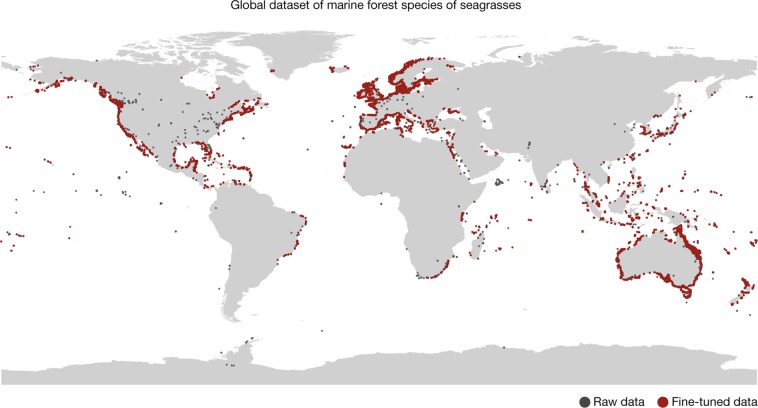
Global dataset of marine forest species of seagrasses. Included families: Cymodoceaceae, Hydrocharitaceae, Posidoniaceae and Zosteraceae. Red and gray circles depict raw and corrected data, respectively.

The dataset provided^41^ covers 682 accepted taxa (at the species level; Suppl. Table 2) belonging to the orders Fucales, Laminariales and Tilopteridales (i.e., brown macroalgae; Fig. 1), and the families Cymodoceaceae, Hydrocharitaceae, Posidoniaceae and Zosteraceae (i.e., seagrass; Fig. 2).

### Spatial and temporal coverage

**Fig. 3:**
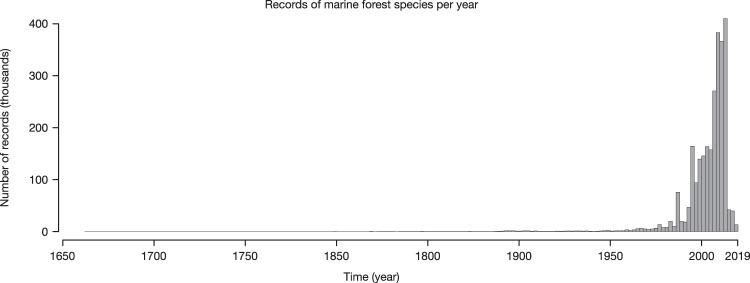
Records of marine forest species per year.

**Fig. 4:**
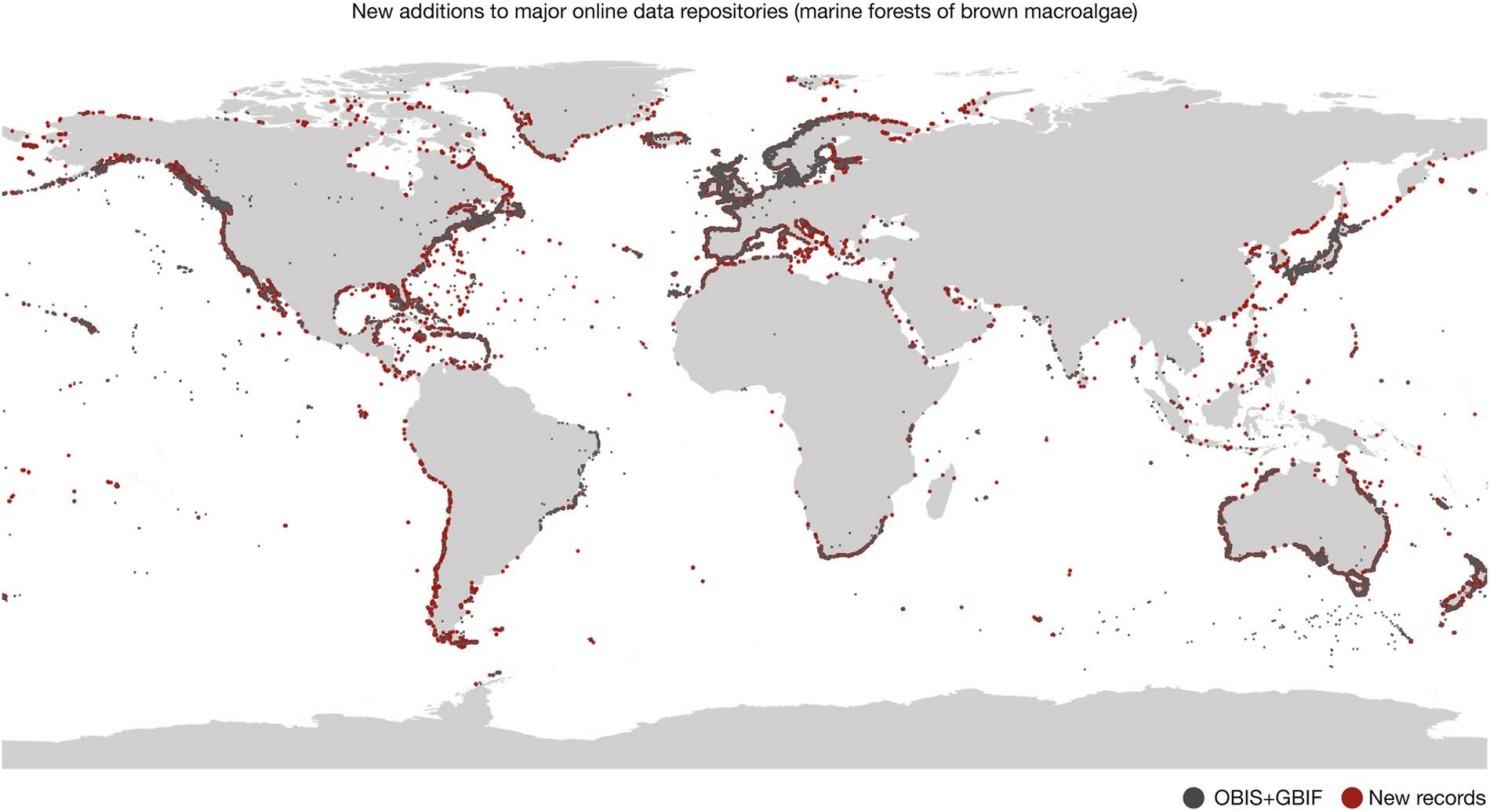
New additions to major online data repositories (marine forests of brown macroalgae). Red circles depict new data and gray circles depict data aggregated from the repositories Global Biodiversity Information Facility^62^ and the Ocean Biogeographic Information System^63^.

**Fig. 5:**
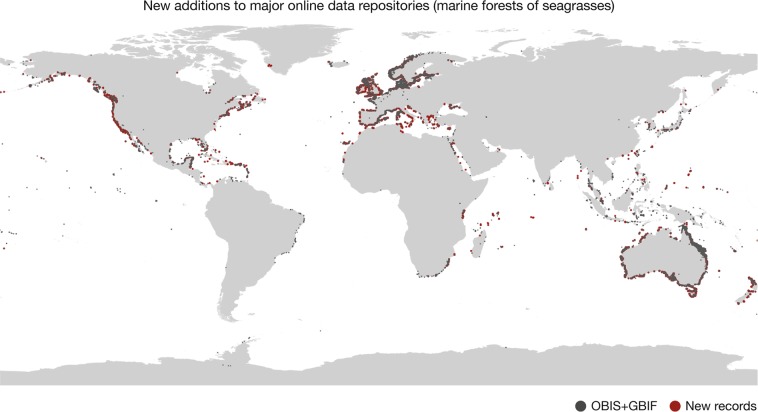
New additions to major online data repositories (marine forests of seagrasses). Red circles depict new data and gray circles depict data aggregated from the repositories Global Biodiversity Information Facility^62^ and the Ocean Biogeographic Information System^63^.

The dataset contains 2,751,805 records of occurrence (brown algae: 1,088,448; seagrasses: 1,663,357; Table 1) globally distributed between the years 1663 and 2018 (Fig. 3), increasing by 47.43% the data available in the two major online repositories GBIF and OBIS (Figs. 4 and 5).

**Table 1:** Summary of records included in the dataset per ecological group, original source type and quality flagged (considering locations on land, regions with unsuitable light conditions and outside known distributional ranges).

Group	Records number (percentage)	Literature	Herbaria	Repositories	Total
Kelp and fucoid algae	Overall	439,877	36,775	611,796	1,088,448
	Flagged: On Land	2,241 (0.51)	5,350 (14.54)	18,615 (3.04)	26,206 (2.41)
	Flagged: Unsuitable light	21,080 (4.79)	7,420 (20.17)	44,480 (7.27)	72,980 (6.70)
	Flagged: Outside distribution	1,013 (0.23)	1,367 (3.71)	4,537 (0.74)	6,917 (0.63)
Seagrasses	Overall	2,376	622	1,660,359	1,663,357
	Flagged: On Land	60 (2.52)	233 (37.45)	6,676 (0.40)	6,969 (0.42)
	Flagged: Unsuitable light	131 (5.51)	254 (40.83)	116,036 (6.99)	116,421 (6.99)
	Flagged: Outside distribution	39 (1.64)	99 (15.91)	68,314 (4.114)	68,452 (4.12)
Total	Overall	442,253	37,397	2,272,155	2,751,805

Values in parenthesis refer to percentage of flagged record.

### Data collection sources

The dataset gathered information from 18 distinct repositories, 15 herbaria and 569 literature sources. The majority of records resulted from external repositories (82.56% of records), followed by literature (16.07% of records) and herbaria (1.35% of records; Table 1). The main repositories GBIF and OBIS accounted for 52.57% of all records). In terms of species number, the main sources of data were external repositories, followed by herbaria and literature. These covered 96.77%, 61.14% and 13.04% of species, respectively (Table 2).

**Table 2:** Summary of species included in the dataset per ecological group and original source type.

Group	Species number (percentage)	Literature	Herbaria	Repositories	Total
Kelp and fucoid algae	Overall	80	396	601	623
	Flagged: On Land	50 (62.50)	333 (84.09)	317 (52.75)	314 (50.40)
	Flagged: Unsuitable light	71 (88.75)	336 (84.84)	513 (85.35)	537 (86.19)
	Flagged: Outside distribution	22 (27.50)	235 (59.34)	208 (34.61)	423 (67.89)
Seagrasses	Overall	9	21	59	59
	Flagged: On Land	8 (88.88)	19 (90.48)	50 (84.74)	52 (88.13)
	Flagged: Unsuitable light	9 (100.00)	18 (85.71)	51 (86.44)	52 (88.13)
	Flagged: Outside distribution	2 (22.22)	17 (80.95)	36 (61.02)	39 (66.10)
Total	Overall	89	417	660	682

Quality flags (considering locations on land, regions with unsuitable light conditions and outside known distributional ranges) refer to species with at least one record flagged. Values in parenthesis refer to percentage of species with at least one record flagged.

## Technical Validation

The dataset gathered information from multiple sources, some of which may be automatically interoperable, sharing erratic duplicated data, regardless of the credibility of the source. These data can be used in scientific studies, potentially generating misleading results. To address the challenge, we developed a specific quality control data treatment based on automatic and manual pipelines.

The taxonomic standardization using WORMS discarded any misspelled or no-match entries from the dataset, and aggregated 1116 initial taxa into 682 accepted taxa (at the species level). As new taxa are being described and their current status is constantly changing, WoRMS may not yet contain all updated statuses^42^, however, it is continuously being improved and is considered the best available source for marine taxonomic standardization. Together with the identification of duplicate entries, records missing coordinate information or information regarding species’ distributional ranges, our approach removed 2,676,350 initial entries from the dataset.

The automatic flagging procedure identified 1.21% of records located on land, and an additional 6.88% records without suitable light conditions for photosynthesis (Table 1). The manual verification based on published literature and consulting experts flagged 2.74% of records as potential outliers outside the know distribution of species (75,369 records; Table 1). Considering the three flags implemented, literature records appeared the least biased (unique exception of literature records for seagrasses flagged over land; Table 1), followed by digital repositories and herbaria (Table 2). The number of species flagged by manual verification against known distributional ranges was the lowest for literature (26.96%), followed by repositories (36.96%) and herbaria (60.43%; Table 2).

The flagging system implemented, not available in any of the 33 repositories and herbaria consulted, allowed delivering a fine-tuned dataset of 2,485,534 georeferenced records gathered from multiple sources, with no taxonomic errors (based on the WoRMS current information), no duplicate entries, no records in unsuitable habitats (i.e. land or low light conditions) or too distant from species’ biogeographical ranges.

The use of a flagging system allowed retaining valuable data that should not be discarded. For instance, some large brown algae and seagrasses can often be found as rafts^43^, floating on the sea surface, hundreds of kilometers away from their original source^44,45^. While these records are not particularly suitable to build ecological models aimed for benthic species, they are highly valuable to address dispersal ecology. Instead of considering such cases as outliers for exclusion, flagging allows keeping records for users to decide their final use.

The dataset will continue to receive new data records from its multiple sources, as new literature gets published and new observations are made. Taxonomic and error corrections will continuously be made over the years, from experts (ecologists, taxonomists and naturalists), allowing continuous flagging of doubtful records.

### R functions for data management and visualization

In addition to the dataset, we developed a set of functions in R language (R Development Core Team, 2018) to facilitate extraction, listing and visualization of occurrence records (e.g., function to export data as geospatial vectors for geographic information systems). All functions are detailed in Table 3 and can be easily installed by entering the following line into the command prompt:

**Table 3:** List of functions available to facilitate extraction, listing and visualization of occurrence records (refer to main Github repository for more information).

Function	Description	Arguments
extractDataset()	Imports data to R environment	group (character), pruned (logical)
listTaxa()	Lists available taxa	—
listData()	Lists data available in a dynamic table	extractDataset object name (character), taxa (character), status (character)
listDataMap()	Lists data available in a map	extractDataset object name (character), taxa (character), status (character), radius (integer), color (character), zoom (integer)
subsetDataset()	Subsets available data to a specific taxon	extractDataset object name (character), taxa (character), status (character)
exportData()	Exports available data to a text delimited file or shapefile (geospatial vector data for geographic information systems)	extractDataset object name (character), taxa (character), status (character), file type (character), file name (character)

source(“https://raw.githubusercontent.com/jorgeassis/marineforestsDB/master/sourceMe.R”).

## Usage Notes

The dataset follows the FAIR principle of Findability, Accessibility, Interoperability and Reusability of data^46^. It is made available as two distinct files in tabular format. The first aggregates all data with no taxonomic errors and no duplicate entries and includes the three fields implemented to flag records. The additional file provides a pruned version of the dataset discarding all potentially biased records.

The dataset complies with Darwin Core Standard (DwC)^32^, providing information on taxonomy, geographical location (e.g., coordinates in decimal degrees, depth and uncertainty), reference to original sources (including permanent identifiers; bibliographic Citation DOI), as well as the flagging system implemented (Table 4).

**Table 4:** Description of the main fields used in the dataset.

Field	Description
id	An identifier given to the occurrence at the time it was recorded
modified	The most recent date-time on which the resource was changed
basisOfRecord	The specific nature of the data record
aphiaID	Unique identifier of a taxon
acceptedAphiaID	Unique identifier of an accepted taxon
name	Taxon’s name, as reported originally
acceptedName	Accepted name’s taxon
scientificNameAuthorship	Name of who described the taxon originally
taxonomicStatus	The status of the taxon (e.g., accepted/not accepted)
kingdom	Higher taxonomic classification
phylum	Higher taxonomic classification
class	Higher taxonomic classification
order	Higher taxonomic classification
family	Higher taxonomic classification
genus	Higher taxonomic classification
decimalLongitude	Geographical longitude in decimal degrees of the center of a location
decimalLatitude	Geographical latitude in decimal degrees of the center of a location
coordinateUncertaintyInMeters	Distance from decimalLatitude and decimalLongitude that describes the smallest circle containing the entire Location
depthAccuracy	Depth uncertainty, as reported originally
country	Country or major administrative unit in which the Location occurs
locality	The specific description of the place
verbatimDepth	Depth in meters
minimumDepthInMeters	Minimum depth in meters
maximumDepthInMeters	Maximum depth in meters
year	The four-digit year in which the Event occurred
month	The two-digit month in which the Event occurred
day	The two-digit day in which the Event occurred
sourceType	Type of original data source
bibliographicCitation	Reference for the resource indicating how this record should be cited
bibliographicCitationDOI	Permanent identifier for the original resource
flagHumanCuratedDistribution*	Flag for records outside the known distribution of species
flagMachineOnLand*	Flag for records occurring over landmasses
flagMachineSuitableLightBottom*	Flag for records outside regions with suitable light conditions
RecordNotes	Additional comments or notes

For more information on additional available fields please refer to the Darwin Core Standard^32^ permanent repository^34,64^ at www.dwc.tdwg.org.

^*^Potentially flagged records as ‘−1’ in dataset.

The integration of the dataset with a set of functions in R language allows easy data acquisition and smooth integration with already available statistical tools, such as those aiming for Ecological Niche Modeling^47,48^. For instance, the dataset can be used to describe the global distribution of species^12,49^, address niche-based questions^3,50,51^, support biodiversity and ecosystem-based conservation^10,52,53^, and to understand correlations between anthropogenic pressures and population extinctions^54^. Additionally, the availability of standard data layers delivering past and future climate change scenarios^36,55^ may further expand the applications of this dataset to predict range shifts^9,56,57^ or hypothesize important evolutionary scenarios, such as mapping climate-refugia where higher and endemic biodiversity evolved^43,58,59^.

Data transparency and accuracy is a prerequisite for avoiding flawed and/or misleading conclusions, especially when provided to stakeholders and decision makers. The pipelines implemented are explicit, ensuring the clarity and reproducibility of the process and contributing to public data in standard formats (i.e., the Darwin Core Standard). With the flagging system, users can fine-tune the original dataset according to their research needs and boost the quality of their results. Particularly, when requested by decision-makers, more accurate outcomes may provide important climate change-integrated conservation strategies^60^, as well as feed important baseline assessments, like those required in the scope of the Intergovernmental Science-Policy Platform on Biodiversity and Ecosystem Services (IPBES).

## Supplementary information

Suppl. Table 1. List of data sources

Suppl. Table 2. List of taxonomic coverage

## Data Availability

Data management was performed using R computing language^61^. The functions developed to manage and flag the dataset are permanently available in a Github repository (https://github.com/jorgeassis/marineforestsDB).
